# Common Bacterial Infections in Persons Who Inject Drugs

**DOI:** 10.3390/medicines12020008

**Published:** 2025-03-28

**Authors:** Michael P. Lorenzo, Kathleen K. Adams, Seth T. Housman

**Affiliations:** 1 Baystate Medical Center, Springfield, MA 01199, USA; michael.lorenzo@baystatehealth.org; 2 School of Pharmacy, University of Connecticut, Storrs, CT 06269, USA; kathleen.adams@uconn.edu; 3 College of Pharmacy and Health Sciences, Western New England University, Springfield, MA 01119, USA

**Keywords:** infections, opioid use disorder, endocarditis

## Abstract

Opioid use in the United States has increased dramatically. Bacterial infections are common among persons who inject drugs (PWID), and there is a disparity in the care these individuals receive. As such, outcomes associated with these infections can be poor. Healthcare providers can address these disparities through optimal pharmacotherapy recommendations and assistance with changing approaches to the management of PWID.

## 1. Introduction

Over the past two decades, the prevalence and landscape of opioid use in the United States (U.S.) have changed dramatically. Drug overdose deaths quadrupled in the U.S. from 1999 to 2019 and almost 500,000 deaths occurred in the U.S. from 1999 to 2019, with opioids contributing to a large portion of these deaths [1]. While the impact of the directly attributable mortality is staggering enough, there are additional contributions to morbidity and mortality among patients with opioid use disorder (OUD) who are also persons who inject drugs (PWID). About 6.6 million people in the U.S. above the age of 13 are PWID, with between 0.5 and 1 million of those with past-year intravenous drug use (IVDU) [2]. While the impact of the opioid epidemic on the healthcare system is vast, with many issues being outside the scope of this review, intravenous drug use carries a significant risk for a variety of infections. The aim of this review is to provide clinicians with current data and their applicability to this population as well as to review novel applications of anti-infectives with specific focus on PWID. The goal is that this information may help to better serve PWID with bacterial infections.

## 2. Barriers to Successful Outcomes

**Figure 1:**
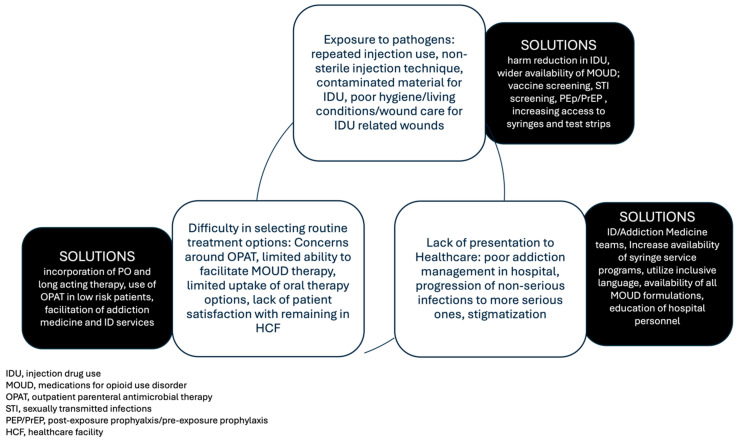
Factors Contributing to complications in management of intravenous drug use-related infections.

Infection-related outcomes in patients with IVDU are generally poorer than that of similar infections in patients without IVDU as a contributing comorbidity. With a particular focus on long-term outcomes, patients with IVDU-related endocarditis have a long-term survival expectation rarely exceeding 60% after 5–10 years or sooner in some examples [3,4,5]. Figure 1 describes factors and potential solutions contributing to infectious complications associated with PWID. Stigmatization by the healthcare system may cause delays in when patients seek care. This may result in patients presenting with more advanced illness and, subsequently, more lengthy and complicated treatment plans, which may be challenging for this patient population [6,7,8]. Within the hospital, research has found that patients with OUD experience different care than patients with similar infections who do not use opioids [9]. A nationwide observational study aimed to evaluate differences in U.S. hospitalizations for serious infections in patients with and without OUD. The study evaluated 95,470 estimated hospitalizations during the year 2016. The length of stay for patients with OUD was 4 days longer than their non-OUD counterparts [9]. In addition, patients with OUD were more likely to leave against medical advice. Patients with OUD were more likely to be sent to a post-acute care facility as opposed to be discharged home. Daily hospital charges were lower for patients with OUD; however, the total hospitalization cost was similar given the extended inpatient stay [9]. Longer hospitalizations can lead to increased rates of nosocomial infections, and leaving against medical advices carries a significant risk of readmission and mortality [10,11]. Multiple factors likely contribute to these outcomes, and barriers to success are present across a number of settings including both inpatient and outpatient practice.

## 3. Disparities in Antimicrobial Team Resources

Blood-borne viruses, namely hepatitis C and the human immunodeficiency virus (HIV), are in the limelight as illustrated by advertisements on television and on social media for the associated medications. In contrast, similar attention is not applied to acute and chronic bacterial and fungal infections. Unfortunately, this discrepancy is not due to a lesser impact of bacterial and fungal infections, but is likely due to lack of awareness.

The differences between the infectious complications related to viral transmission versus bacterial and fungal infections contrast in many ways. The relative frequency of such infections differs drastically, with injection site infections far exceeding rates of chronic viral infection in patients with IVDU, and more serious infections such as bacterial endocarditis competing with regard to prevalence of IVDU-related transmissions of hepatitis C and HIV [12]. The chronicity and acuity of such viral and bacterial infections also differ with few patients with IVDU-related viral infections presenting to hospitals for initial diagnosis or treatment of said infection. Patients with IVDU-related bacterial infections may not seek care for their infections and subsequently require an inpatient admission or emergency department evaluation if not linked to routine care [13].

There exists a disparity in the care and resources available for PWID with chronic viral infections vs. PWID with bacterial and fungal infections. Healthcare institutions have invested resources in ensuring optimal clinical outcomes for patients with HIV and hepatitis C by establishing specialty clinics [14]. In the case of a PWID contracting hepatitis C, an interdisciplinary approach is utilized due to the complexity of pretreatment workup, insurance authorizations, and coordination of care to ensure completion of therapy, which may include directly observed therapy. Such patients will often be managed by an individual or even a multidisciplinary team with specialized training in the management of this disease and may include a pharmacist to counsel and monitor care [14]. This comprehensive approach to care is something that patients presenting with bacterial or fungal infections may not receive. Patients hospitalized for an acute bacterial or fungal infection are likely cared for by a generalist who may have an inadequacy of training to optimally treat infections, and these patients may or may not have the opportunity to be evaluated by an infectious disease specialist [15,16,17]. This potential lack of experience of treating infectious diseases coupled with the lack of clinician preparedness to manage co-occurring substance use disorders creates a scenario where such patients are at a risk of treatment failures, incomplete courses of therapy, and high rates of recurrent infections [18,19]. While improvement in antibiotic decisions in PWID will not completely solve the mortality and morbidity associated with infectious complications of IVDU, there is an opportunity to improve this aspect of care.

### 3.1. Lack of Access to Addiction Medicine Specialists

Not all patients will receive appropriate management of opioid use disorder while being treated for their infection. Prolonged admissions can be complicated by opioid cravings and withdrawal if not managed properly, and this may result in patients leaving against medical advice (AMA) prior to completing potentially optimal antimicrobial therapy [18]. A 2019 retrospective observational study by Marks and colleagues found that patients seen by an addiction medicine specialist during their inpatient admission were more likely to complete antibiotic therapy and less likely to be discharged against medical attention or elope from the hospital [19]. Additionally, this study found that patients seen by the addiction medicine service had fewer readmissions within 3 months after discharge. A 2020 retrospective cohort study by Tan and colleagues found that addiction medicine treatment was associated with a significantly lower rate of new bloodstream infections [20]. It has been shown that using replacement therapy, such as buprenorphine, to treat opioid use disorder may increase adherence to treatments for unrelated conditions [21]. This again suggests that addiction management should be co-managed with antimicrobial therapy in this patient population. Clinicians should utilize the inpatient admission for treatment of infection as an opportunity to maximize current opioid use disorder pharmacotherapy and consult their addiction medicine service if assistance is required.

### 3.2. Outpatient Parenterial Antimicrobial Therapy Hesitancy

Outpatient parenteral antimicrobial therapy (OPAT) was originally described in the United States in the 1970s when patients with cystic fibrosis received intravenous antibiotics in the community setting [22]. Since then, OPAT has emerged as an effective method for delivering parenteral antimicrobial therapy. Venous access for administration of parenteral antibiotics is an essential aspect of OPAT. Access is routinely achieved with the placement of a peripherally inserted central catheter (PICC), and the PICC remains in place for the duration of the treatment. Some guideline groups and institutions have deemed PWID poor candidates for OPAT due to the risk of PICC misuse, and some survey studies have illustrated low acceptance of OPAT for PWID among infectious disease physicians [22]. The validity of this hesitancy may be unjustified.

A 2018 literature review by Suzuki and colleagues found that 72% to 100% of PWID successfully completed OPAT, which is similar to outcomes in patients who do not inject drugs [23]. This success rate is estimated to be 80–90% globally. In addition, mortality for PWID in the review ranged from 0% to 10.3% with 7 of 10 studies reporting no deaths in their PWID cohort. This may be only slightly higher than mortality in patients without a history of injection drug use of 0.1–0.4%. Line-related complications and OPAT completion rates were generally comparable among PWID and non-PWID cohorts; however, re-hospitalization rates may be increased among PWID. One study was identified that documented a PICC misuse rate of 2%. While more information is needed, preliminary data suggest that there may be cost savings with OPAT in PWID. Finally, in a 2020 retrospective cohort study by Tan and colleagues that analyzed 420 unique episodes of infective endocarditis among 309 PWID, outpatient treatment was not associated with an increased rate of new bloodstream infections or an increase in mortality [20].

Clinicians should consider general OPAT guidelines to assess if patients could be appropriate candidates. If a patient is considered stable and ready for discharge, a number of aspects need to be in place to facilitate successful OPAT [24]. Providers should ensure that the patient’s place of discharge is supportive and safe. The place of discharge must also be able to facilitate successful medication therapy. This includes a fixed address, a refrigerator and electricity for medication storage, running water for sanitation purposes, and telephone service to reach OPAT staff if needed. PWID that are homeless remain at high risk for loss to follow-up, secondary bacteremias, and 30-day readmissions [25]. As a result, facilitating temporary housing may be the optimal choice in this patient population. The patient and/or caregiver should be capable of safe medication administration and must be willing to participate in the patient’s care. In addition, the patient and/or caregiver must be physically and mentally able to manage the prescribed treatment protocol. Therapeutic monitoring can be established whether in a clinic or at home, and the patient needs to accept financial responsibility of costs not covered by insurance.

More specifically, a nine-point scale developed by Eaton and colleagues can provide clinicians with a risk stratification tool to decide what patients are “low risk” and are likely to be more successful if discharged on OPAT [26]. The scale takes into account cravings, stability of the discharge environment, concurrent psychiatric comorbidities, history of drug overdose and relapse, concurrent polysubstance abuse, family history of addiction, history of trauma, and willingness to change. While limiting intravenous access in PWID is likely well intentioned by clinicians, the concern may not be clinically sound. In the correct patient, OPAT can be a feasible option for PWID and may result in cost-saving opportunities in addition to improved clinical outcomes.

### 3.3. Opioid Impact on Immune Response

In addition to their role in analgesia, addiction, and respiratory depression, opioids interact with opioid receptors on the cell membrane to play an important role in physiological and pathophysiological processes of the immune system [27]. A suboptimal immune response can result in a lack of protection and worsen disease severity. Chronic use of opioids has been shown to compromise the immune system, leading to an increased risk of opportunistic infections. Both the innate immune response and the adaptive immune response are critical aspects of the body’s immune system. Chronic opioid use, specifically morphine, has been shown to impact both of these immune responses. Morphine impairs proliferation of macrophage cells and inhibits the secretion of cytokines resulting in reduced chemotaxis. While factors such as sharing contaminated needles, nutrition, environment, history of illicit drug use, and genetics impact infection frequency in this group of patients, studies suggest that opioids may also have immune-modulating impacts.

### 3.4. Changing Approach to Management of Infection

Historically, guidelines for the management of endocarditis have previously recommended oral antibiotics in few scenarios and have mainly recommended agents from only two antibiotic classes as the backbone of therapy (fluoroquinolones and oxazolidinones), with European guidelines allowing for slightly more latitude in oral treatment options [28,29]. Osteomyelitis and native joint infections, without unifying international guidelines, are subject to more variability in treatment decisions, but historically have been treated with high rates of OPAT utilization [30,31]. Gram-negative bacteremia is also subject to a high degree of variability in therapy with treatment durations often ranging from 7 to 21 days, a variety of oral and intravenous antibiotics used for therapy, and guidance on treatment duration commonly coming from the guideline for the management of central-line-associated bacteremia [32,33,34]. Within the past few years, there have been several articles published that may serve to fuel a dramatic change in the management of such complicated infections.

The POET, OVIVA, and a trial conducted by Yahav et al. have all challenged long-held treatment standards for the treatment of endocarditis, bone and joint infections, and Gram-negative bacteremia, respectively [35,36,37]. While these data will likely impact antibiotic-prescribing patterns in their respective fields, of the 2000 patients included between these three trials, there are only 5 IVDU reported in total. This creates an opportunity where this robust data may not be used when designing treatment regimens for patients with IVDU. For example, the treatment regimens in the POET trial were quite complex. The lowest amount of administrations per day was 2, with 2 separate oral antibiotics being given at 2 different points during the day. The most complex regimen was 2 different drugs being given 4 and 2 times a day concurrently. This may be feasible for many patients, including those patients who are more stable in their addiction and able to reliably self-administer antibiotics. However, patients with unstable housing or ability to store medications, concurrent psychiatric disorders, or patients who have previously shown noncompliance with therapy for OUD or other chronic comorbidities may be unlikely to adhere to these regimens. Also, there was a high rate of surgical management of endocarditis in this trial, which is unlikely to be a trend reflected in many patients with a history of IVDU. This, along with a difference in expected causative microorganisms from what is observed in IVDU-related endocarditis in the U.S., may further limit applicability [35,38,39]. Finally, there was a routine use of therapeutic drug monitoring, which serves as a direct limitation in applicability. Encouragingly, the available PK data from this study would suggest future need for therapeutic drug monitoring using their dosing regimens is unnecessary. Alternatively, the trial by Yahav et al., while not detailing the rate of OUD or IVDU in either treatment group, may share more applicability to this population because of the overall short duration of treatment and predominant use of fluroquinolones in the oral therapy arm that could be dosed once daily to complete a 7-day total course [37]. The OVIVA trial involved a common infectious complication of IVDU, reflected a microbiologic etiology similar to what would be expected in an IVDU population, and received antibiotic regimens based on provider discretion. Some characteristics of the patient population may not be as transferable given the high rate of metalware infections unlikely to be present in the younger population of IVDU, and the high rate of compliance with oral therapy (only ~10% of patients evaluable reported <90% compliance) may be cause for some pause in applying these results directly to an IVDU population [36].

While these trials may drive significant practice changes and be incorporated into future international guidelines, they reflect a trend seen in many investigations that are positioned to challenge prior treatment paradigms. All the mentioned trials had low rates of reported IVDU. This is not unexpected given the U.S. was not a recruiting site for any of them. In three recent prospective studies focused on patients with *S. aureus* bloodstream infections, there seems to be an under-representation of patients with substance use or IVDU listed as a comorbidity [40,41,42]. This highlights an overall issue with transferring data from any trial to special populations, but with specific reference to patients reporting IVDU, it may be particularly concerning given the large contribution of this population to cases of *S. aureus* bacteremia at many institutions in the U.S., and the overall poor long-term outcomes displayed in this population. As a result, available data on the management of such infections come from smaller, often retrospective reviews of the management of specific infections in people who are also IVDU. While specific treatment options that seem promising will be discussed below, there is an emerging multidisciplinary approach to management that is worth highlighting. There have been several recent calls for cross training of infectious disease clinicians in addiction medicine to create multidisciplinary teams that have been shown to provide positive outcomes in other infectious complications in IVDU, e.g., IVDU-related infective endocarditis [43,44,45,46]. These efforts may not only provide an expertise in management of infections in these patients but may also serve to expand the understanding and empathy surrounding patients’ struggles with addiction.

## 4. Available Therapies That May Improve Outcomes/Compliance

So far, the major focus of this review has been the utilization of various methods of care to optimize outcomes in IVDU-related infections but a common factor among oral antibiotics, OPAT, and comprehensive team-based approaches to care is the use of anti-infectives. There are emerging data regarding specific anti-infectives that warrant discussion within this context.

### 4.1. Long-Acting Glycopeptides

Long-acting lipoglycopeptides are a recently developed class of antibiotics with two representative drugs, dalbavancin and oritavancin. Both are only approved for skin and soft tissue infections by the Food and Drug Administration but have been studied in small observational trials for multiple more complicated infections. The pharmacokinetics of these drugs are similar, with particularly long terminal half-lives allowing for infrequent administrations and single-dose administrations that represent up to two weeks of therapy. The infrequent need for IV access, obviating the need for a longstanding intravenous catheter, and the limited need for safety or therapeutic drug monitoring make them a safe option for patients unlikely to engage in routine follow-up. The spectrum of activity is nearly identical to vancomycin, the current standard of care for serious infections caused by MRSA [47]. These drugs have quickly developed an obvious role in IVDU populations who may present difficulties in follow-up and have frequently extended courses of intravenous antibiotics.

The pharmacokinetics (PK) and pharmacodynamics (PD) of these drugs are similar, but there have been more data and more novel dosing strategies published with dalbavancin to date [48,49,50]. Both drugs display PD consistent with antibacterial activity determined by the ratio of the area under the concentration time curve to minimum inhibitory concentration (AUC:MIC), have established breakpoints for most clinically relevant Gram-positives, display activity against >90% of Staphylococcal isolates in the U.S., and have single-dose regimens approved for the treatment of complicated skin and soft tissue infections [48,49,50].

Research on repeated and long-term dosing of oritavancin is sparse and inconsistent. Table 1 describes dosing strategies for long-acting glycopeptides from the selected literature. The first clinical experience with repeated dosing is represented by a case report which utilized a front-loading dosing strategy (1200 mg every 48 h for 3 doses) followed by weekly doses of 1200 mg for 6 weeks in the treatment of a prosthetic valve endocarditis caused by a vancomycin-resistant Enterococcus faecium [51]. This dosing strategy provided a positive clinical outcome in a difficult case but provides little generalizable information for a few reasons. The isolate in this case was an E. faecium with a reported MIC of 0.5 mcg/mL which is 2-fold higher than the CLSI-approved breakpoint for vancomycin susceptible E. faecalis, and 3-fold higher than the MIC90 of over 9000 clinical isolates of *S. aureus* [50,52]. Other case reports utilized weekly dosing strategies of 1200 mg but were not treating such a challenging isolate and patient case [53,54]. The most clinical experience with repeated dosing of oritavancin comes from the CHROME study which reported a wide array of dosing strategies reflective of provider preference, making it difficult to conclude that the choice of one dosing strategy is optimal [55]. A more recent pharmacokinetic analysis utilizing an initial 1200 mg dose with subsequent dosing of 800 mg after a week shows promise as a treatment option for long-term therapy. While this analysis only utilized a single repeat dose, there is unlikely to be accumulation beyond safe levels as seen by the safety reported in the Johnson case report which utilized much higher dosing and goal concentrations or a decrease below therapeutic concentrations with dosing beyond what was utilized [56]. Similarly, a small number of patients in a case series of IVDU received this oritavancin dosing strategy, and good rates of overall clinical success were reported. This series, along with a single patient with reported history of IVDU who received 7 weeks of 800 mg/week following a 1200 mg loading dose, represents the cumulative reported experience of this agent in the management of infections in patients who are PWID [57,58].

In contrast to oritavancin, there does seem to be more experience with long-term use, clearer dosing requirements, novelty in dosing strategies, and use in PWID with dalbavancin. Due to the longevity of experience with dalbavancin in BSIs dating back to as far as 2005 in a trial evaluating the use of dalbavancin for catheter-related BSIs, there have been more published data for dalbavancin in both serious infections and consistency in overall dosing scheme with most trials utilizing weekly or bi-weekly dosing with 500 mg or 1000 mg following loading doses of 1000 mg or 1500 mg, respectively [59,60,61,62]. Importantly, with dalbavancin, there have been several investigations published that specifically focus on PWID or patients with significant social barriers to care [63,64,65,66]. The cumulative experience with dalbavancin in this population is encouraging with cure rates similar to patients able to complete therapy and a logistical benefit to increasing rates of therapy completion, though this has not been clearly demonstrated to date, as none of the published literature focuses on a change in completion rates in therapy with dalbavancin compared to historical institutionally specific rates. Institutional data suggest treatment completion is similar between patients receiving dalbavancin versus non-dalbavancin regimens for the treatment of *S. aureus* bacteremia with 19/29 and 11/20 patients, respectively, completing treatment [67]. Importantly, Morrisette et al. demonstrated a cost-savings benefit with the use of dalbavancin, though it did not reach statistical significance due to low overall patient numbers in the trial [66]. This is one of the major concerns with the use of dalbavancin, as the current average wholesale price for a single 500 mg vial is USD 1953 [68]. A patient would need anywhere from 2 to 7 vials based on the required treatment duration and pretreatment prior to dalbavancin initiation to complete a 6-week course of therapy. Despite the high cost, a trend towards being cost-beneficial may encourage more institutions to be willing to develop pathways for the use of this class of drugs. One of the most exciting dosing strategies using dalbavancin may be the use of a two-dose regimen, of 1500 mg on day 1 and day 8, representing 6 weeks of therapy [69]. This strategy, initially studied and shown to be safe and efficacious in a phase II study for osteomyelitis, may represent a truly novel drug delivery pathway for PWID with IE given the cumulative evidence of dalbavancin in IE and the high serum concentrations seen throughout this dosing interval, which is likely to provide an appropriate PK/PD exposure for the entirety of a treatment course for IE [69,70].

### 4.2. Oxazolidinones

Linezolid, and more recently, tedizolid are two drugs with a broad Gram-positive spectrum that may prove to have significant utility in treating serious infections related to IVDU. The high degree of bioavailability, manageable oral administration schedule, robust activity against *S. aureus*, and familiarity with use in severe infections caused by MRSA makes these drugs alternates to OPAT and worth consideration in certain patient populations, including PWID. Linezolid has demonstrated efficacy as step-down therapy (from IV therapy) in uncomplicated *S. aureus* bloodstream infections, and while predominately represented by MSSA presumably in a non-IVDU population, Willekens et al. outline a promising pathway for the use of PO linezolid in appropriate patients [71]. With specific reference to PWID, a recent retrospective study reflecting usual practice at an ID clinic in Detroit noted linezolid to be the most commonly utilized oral agent in the management of MRSA BSIs with roughly one-third of the population receiving oral antibiotics reported to be IVDU [72]. Notably, this investigation noted no difference in 90-day clinical failure rates and a decrease in 90-day hospital readmission in the oral antibiotic arm. Tedizolid has no published data for use in PWID and limited data for use in bloodstream infections as a whole [73]. Limited efficacy data in this population and the high overall cost make in-depth discussion of this drug of limited relevance.

It would be a logical conclusion to use linezolid for the oral therapy for many Gram-positive BSIs in PWID, but there may be several misgivings regarding this practice. Linezolid historically has been used rather sparingly for BSIs as a whole, mainly due to initial concerns over efficacy in this setting and continued concerns over safety that have largely relegated this drug to being an alternate to vancomycin in many cases. Initial approval of linezolid was followed shortly by several black box warnings. One of the initial safety concerns came from the results of a phase 3 trial investigating the use of linezolid in central-line-associated bloodstream infections, in which an imbalance of mortality was seen with a higher distribution in the linezolid arm [74]. This imbalance is primarily due to involvement of Gram-negative and culture negative episodes, which were possibly due to organisms for which linezolid would not be an appropriate option. Safety concerns also arose surrounding the inhibition of monoamine oxidase (MAO) leading to serotonin toxicity in patients on additional serotonergic agents; this may be particularly concerning in PWID due to a reported interaction with methadone and fentanyl, which are becoming more prevalent in heroin supplies [74]. Finally, providers may be uncomfortable prescribing extended courses of linezolid to patients who may be unwilling or able to participate in routine follow-up, due to longer courses being associated with thrombocytopenia; while these are all valid concerns, emerging data may serve to mitigate some prescribers’ fear of using this drug. Serotonin toxicity is a possible adverse effect of any drug that interacts with production of precursory neurotransmitters or the metabolism of serotonin itself, and while it is logical to consider linezolid to increase the risk of this toxicity due to its inhibition of MAO, comparative data with historically non-serotonergic drugs have displayed no such risk with linezolid in two retrospective studies [75,76]. Comparative data may be of more value to elucidate the risk of this drug interaction, rather than the commonly cited case series and reports that are unable to give the reader context to the baseline frequency of this event in a control group. With regard to thrombocytopenia related to longer courses of linezolid, more recent data would suggest that a strong driver for this adverse event is decreased clearance of linezolid resulting in supratherapeutic concentrations [77]. It is a fortunate circumstance for the utility of this drug in PWID that many of these patients may be young, with limited comorbidities, and without decreased renal clearance of most drugs, and while normal renal function does not completely mitigate this risk, the decreased occurrence of thrombocytopenia in this population may provide more comfort in prescribing this drug to treat serious Gram-positive infections in PWID [77,78].

### 4.3. Other Oral Agents

Oral antibiotics are likely to serve a large role in treating many infections in IVDU as previously discussed; however, the amount of literature surrounding the use of any single drug in PWID is scarce outside of the options discussed previously. Current trials seem to be lacking with only one study identified on clinicaltrials.gov when searching for PWID and oral antibiotics [79]. Current guidelines do recommend the combination of a fluoroquinolone with rifampin for the treatment of right-sided staphylococcal IE in patients unable to receive IV antibiotics, with this recommendation being guided by two studies that supported the use of this combination in PWID [80,81]. The use of rifampin in this regimen presents issues with decreasing concentrations of methadone and buprenorphine, which could lead to withdrawal symptoms and be counterproductive in the management of infections related to IVDU [82,83]. This strategy may also be limited to use in BSIs or IE caused by MSSA, due to the frequency of resistance in MRSA. Oral fluoroquinolones alone, however, are a well-established agent as monotherapy for Gram-negative bloodstream infections [32,33]. Oral beta lactams and oral trimethoprim/sulfamethoxazole do seem to be promising options for the treatment of Gram-positive and Gram-negative BSIs alike; there is a need for clarity with respect to the appropriate dosing of these agents for BSIs and possible IE, and success rates in PWID given the frequent need for administration (particularly with beta lactams) [33,35,72,84].

## 5. Conclusions

The opioid epidemic shows little sign of abating in the near future, and while the training of healthcare professionals does show signs of adapting to this challenge, addiction medicine fellowships, pain management pharmacy residency programs, and increased access to buprenorphine all reflect an increasing desire to serve patients afflicted by OUD [85,86,87]. While these are all important strides, there will be a continued need to address the collateral damage provided by the opioid epidemic. Just as increased awareness, training, and understanding of treatment will serve to more appropriately manage the grip of addiction, an adjustment of antimicrobial prescription in PWID may serve to mitigate the risk associated with therapy, increase treatment completion rates, and decrease the morbidity associated with infectious complications. The joining of these two strategies may be of benefit to a significant number of patients. Furthermore, future research is desperately needed on a multi-disciplinary approach to the management of patients with infections and the use of novel therapies for infections within this patient population.

## Figures and Tables

**Table 1:** Selected dosing strategies for long-acting lipoglycopeptides.

Drug	Dosing Recommendations	Duration of Therapy	Reference
Oritavancin	1200 mg IV once	7 days	[55]
	1200 mg IV every 48 h × 3 doses, followed by 1200 mg weekly	Weekly *	[51]
	1200 mg IV every week	Weekly *	[53,54]
	1200 mg IV on week 1, followed by 800 mg IV weekly	Weekly *	[57,58]
	1500 mg IV once	14 days	[59]
Dalbavancin	1000 mg IV followed by 500 mg weekly, one week later	Weekly *	[60]
	1500 mg IV followed by 1000 mg every other week, two weeks later	2 weeks **	[59,61,62]
	1000 mg IV every 14 days	Weekly *	[59]
	1500 mg IV followed by 1500 mg 8 days after	42 days	[59]

* weekly dosing can extend duration for 7 additional days. ** extends duration by two weeks for every dose.

## Data Availability

No new data were created or analyzed in this study. Data sharing is not applicable to this article.
